# Characterization of cases and analysis of First-instance Judgments in Ethical Proceedings at the Regional Council of Veterinary Medicine of the State of São Paulo

**DOI:** 10.29374/2527-2179.bjvm002725

**Published:** 2025-10-07

**Authors:** Roberta Tognareli Ruiz, Rosemary Viola Bosch, Suely Stringari, Jorge Luis Maria Ruiz, Paulo Cezar Maiorka

**Affiliations:** 1 Programa de Pós-graduação em Patologia Experimental e Comparada. Universidade de São Paulo. SP, Brazil; 2 Laboratório de Biotecnologia Aplica à Saúde. Universidade Federal da Integração Latino-Americana -UNILA, Foz do Iguaçú, PR, Brazil

**Keywords:** Ethical code, Ethical process, case law, CRMV-SP, Código de ética, Processo ético, jurisprudência, CRMV-SP

## Abstract

In Brazil, veterinary medicine is regulated by a resolution that sets out the Code of Ethics of the Veterinary Medical Profession, established by the Federal Council, which has national jurisdiction and compliance with which is monitored by the regional councils in each state. Some regional councils have reported an increase in the number of complaints of unethical proceedings in recent years, although this trend is not uniform across all Brazilian states. This increase has not yet been consistently documented. In this paper, a quantitative and qualitative description of cases of ethical infractions reported to the Regional Council of Veterinary Medicine of the State of São Paulo—the largest in Brazil, with approximately 59,324 (29%) registered veterinary doctors—is presented, covering the period from 2021 to 2023. Thus, a retrospective study was carried out to determine the profile of the reported professionals, the main motivations for the ethics-based complaints, and to characterize the case law of the cases judged in the first instance at the Regional Council of Veterinary Medicine of the State of São Paulo (CRMV-SP). The data analyzed shows that half of the complaints in the period were considered unfounded, and 123 complaints resulted in different types of conviction. The main article violated in the analyzed cases is Article 9 of the Code of Ethics for Veterinarians, which addresses ethical responsibility in the provision of professional services. This study demonstrates the need for veterinarians to become better acquainted with the ethical standards of the profession.

## Resumo

No Brasil, a medicina veterinária é regulamentada por uma resolução que estabelece o Código de Ética da Profissão Médico-Veterinária, elaborado pelo Conselho Federal, que possui jurisdição nacional e cujo cumprimento é fiscalizado pelos conselhos regionais em cada estado. Alguns conselhos regionais relataram um aumento no número de denúncias de condutas antiéticas nos últimos anos, embora essa tendência não seja uniforme em todos os estados brasileiros. Esse aumento ainda não foi documentado de forma consistente. Neste trabalho, apresenta-se uma descrição quantitativa e qualitativa dos casos de infrações éticas comunicados ao Conselho Regional de Medicina Veterinária do Estado de São Paulo, o maior do Brasil, com aproximadamente 59.324 (29%) médicos-veterinários registrados, abrangendo o período de 2021 a 2023. Assim, foi realizado um estudo retrospectivo para determinar o perfil dos profissionais denunciados, as principais motivações para as denúncias baseadas em ética e para caracterizar a jurisprudência dos casos julgados em primeira instância no Conselho Regional de Medicina Veterinária do Estado de São Paulo (CRMV-SP). Os dados analisados mostram que metade das denúncias no período foi considerada improcedente, e 123 denúncias resultaram em diferentes tipos de condenação. O principal artigo violado nos casos analisados é o Artigo 9 do Código de Ética do Médico-Veterinário, que trata da responsabilidade ética na prestação de serviços profissionais. Este estudo demonstra a necessidade de que os médicos-veterinários se familiarizem melhor com as normas éticas da profissão.

## Introduction

Morality refers to the set of norms and values that guide human behavior within a society, establishing what is considered correct or incorrect. These principles directly influence professional ethics, which consist of the guidelines and standards of conduct that govern the practice of a profession, ensuring responsible practices in line with social values (Arnsperger & Van Parijs, 2004; Borges & Medeiros, 2007; Conselho Regional de Medicina Veterinária do Estado de São Paulo, 2024b; Tostes et al., 2024).

Cortina & Martínez (2005) stated that professional ethics are related to the norms that form the conscience of the professional and represent imperatives for their conduct. It is also the set of technical and social behaviors demanded of members of a professional community. By acting in accordance with established standards, the professional adopts an expected posture in the performance of their duties, valuing their profession, and adequately serving those who depend on it. Thus, professional codes of ethics are integrated as complementary instruments, standards carefully established by professional councils to regulate and direct each profession, ensuring that professional practice in a given area is guided by well-defined guidelines (Glock & Goldim, 2003). These established guidelines constitute the foundations that direct professionals in the performance of their duties, clearly outlining their permissions and restrictions.

In Brazil, the professional practice of veterinarians is regulated by the Federal Council of Veterinary Medicine (CFMV) and supervised regionally by the Regional Councils of Veterinary Medicine (CRMVs). The CFMV, established by Law No. 5.517/1968 and Decree No. 64.704/1969 (Brasil, 1968, 1969), is responsible for setting nationwide rules, ethics guidelines, and general principles for the profession. It also oversees and validates sanctions in severe cases of professional misconduct. The CRMVs operate in each Brazilian state and are responsible for registering professionals, monitoring compliance with ethical and legal standards, and initiating and judging ethical proceedings within their jurisdiction. These councils apply disciplinary actions when ethical violations are identified, in accordance with national legislation and the Code of Professional Ethics for Veterinarians, currently defined by CFMV Resolution No. 1138/2016 (Brasil, 2016). Resolution 1138/2016 outlines not only the duties and rights of veterinarians and the principles guiding professional conduct, but it also defines how professionals must interact with clients, animals, colleagues, the environment, and society. It also establishes the range of applicable sanctions, which include: confidential warning, confidential censure, public censure, temporary suspension of professional practice (for up to three months), and, in the most serious cases, revocation of the right to practice—which requires approval from the CFMV (Bosch, 2015; Conceição et al., 2017; Conselho Regional de Medicina Veterinária do Estado de São Paulo, 2024a). Therefore, it goes beyond the clinical care of companion animals. It is a profession strategically positioned at the interface between animal health, human health, and environmental health, playing a key role in promoting balance and sustainable development in society.

In Brazil, there has been an increase in the number of complaints of ethical proceedings involving veterinarians, and the Regional Council of Veterinary Medicine (CRMVs) is responsible for imposing penalties proportional to the seriousness of the infractions (Conselho Regional de Medicina Veterinária do Estado de São Paulo, 2024b; Tostes et al., 2024). Reports can be submitted via email or through the official websites of the Regional Veterinary Councils. Faced with this scenario, it is essential that professionals are familiar with the ethics guidelines and follow them rigorously, guaranteeing not only the value of veterinary medicine but also the safety and trust of society. Analyzing this issue in Brazil is helpful in order to understand the increase in complaints and to clarify their causes. This paper presents a retrospective study of complaints made by the Regional Council of Veterinary Medicine of the state of São Paulo, the largest council in Brazil, which currently has approximately 73,000 registered veterinary doctors. This work may serve as a reference for comparing veterinary codes of ethics across different countries and offer valuable insights for enhancing professional practices globally, aligning them with standards recognized for their ethical and technical rigor.

## Material and methods

### Retrospective study

Data for this study was obtained by surveying and analyzing ethical proceedings records in collaboration with the Regional Council of Veterinary Medicine of the State of São Paulo (CRMV-SP). Access to the data was granted by the CRMV-SP board *ad referendum*. The dataset covers the period from 2021 to 2023 and includes detailed information such as the date of receipt of the complaint, type of professional registration of the respondent, age group, sex, academic degree, years since graduation, location of professional activity, prior disciplinary history, professional area of the respondent, identity of the complainant, reason for the complaint, procedural stage, dates of instruction and judgment, decision, and any fines applied. Data were tabulated using Microsoft Excel® and presented using descriptive statistics. Most analyses were conducted with data stratified by year. For quantitative variables, when appropriate, one-way analysis of variance (ANOVA) followed by Bonferroni’s post hoc test was applied. All statistical analyses were performed using GraphPad Prism 8, and statistical significance was defined as P < 0.05.

## Results

**Figure 1:**
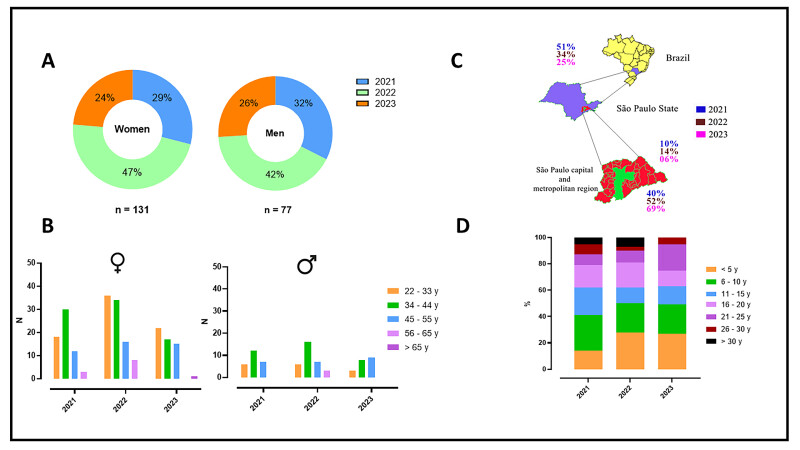
Distribution of genders, age group, geographical region and length of training of those processed.

Note: A- Distribution of the sex of defendants from 2021 to 2023. B- Distribution of age groups between the sexes of the defendants in the years 2021 to 2023. C: Regional distribution of cases. D: Time of formation until the start of the case.

This retrospective study evaluated the processes in the database of the Regional Council of Veterinary Medicine of São Paulo. Data was obtained for 2021, 2022, and 2023. The total number of complaints recorded for each year was 208, with 63, 94, and 51 cases, respectively (Figure 1A). The ethics violations that led to penalties are summarized in Table 1. Because each article of the ethical code includes several sub-items, listing all specific infractions would make the table overly complex and difficult to read. Therefore, we grouped the cases based on the main article used to justify the penalty. It is important to note that the application of penalties may vary depending on how each case was interpreted and on the combination of multiple violations within the same process, which contributes to the heterogeneity observed. No statistical difference was observed in the proportion of defendants by sex when the three years were analyzed together (Figure 1B). However, 2022 showed a marked increase in the number of cases compared to 2021 and 2023, making it the year with the highest number of proceedings initiated. This peak may be related, at least in part, to the residual effects of the COVID-19 pandemic, such as delayed reporting or accumulated complaints. Additionally, it is important to note that the decision to open a proceeding depends on the interpretation and judgment of the Council's president, which may directly influence whether a complaint is accepted. In terms of regional distribution, most complaints originated in the city of São Paulo, the state capital. There was a clear upward trend, with the proportion of complaints from the city of São Paulo increasing from 40% in 2021 to 69% in 2023, as illustrated in the geographical distribution map (Figure 1C) and Figure 1 B—distribution of reported veterinarians by sex and year of complaint (2021–2023). The chart shows that a greater number of reports involved women (n = 131) compared to men (n = 77). Among women, the largest proportion of complaints occurred in 2022 (47%), followed by 2021 (29%) and 2023 (24%). For men, the trend was similar, with the highest proportion in 2022 (42%), followed by 2021 (32%) and 2023 (26%). This pattern suggests a consistent predominance of complaints in 2022 across both sexes, with a slightly higher representation of women among those reported overall.

**Table 1:** Resolution 1138 CFMV articles infringed and used to determine convictions.

**Articles**	**1°**	**2°**	**3°**	**4°**	**5°**	**6°**	**8°**	**9°**	**10°**	**11°**	**14°**	**15°**	**17°**	**25°**	**26°**	**Total**	**Fine**
**Verdict**																	
**Confidential warning in reserved notice**	5	0	1	0	1	0	7	36	1	0	1	1	0	0	1	37	6
**Confidential censure in reserved notice**	8	2	1	1	3	2	28	47	2	1	2	2	5	0	0	50	31
**Public censure in official publication**	3	1	6	1	2	2	33	30	1	0	0	0	2	1	0	28	23
**Suspension from professional practice for up to 3 months**	6	2	4	0	0	1	12	5	0	0	0	0	0	0	0	8	8
**Dismissed**	0	0	0	0	0	0	0	0	0	0	0	0	0	0	0	84	0

Distribution of penalties applied in ethical proceedings according to the article of the Veterinary Code of Ethics (Res. 1138 CFMV) cited in each decision. Penalties are organized by type (confidential warning, confidential censure, public censure, suspension for up to 3 months, and dismissal), and articles are grouped by number, with subsections and items consolidated. The "Fine" column indicates cases in which a monetary fine was applied in addition to the main penalty. The "Total" column shows the total number of occurrences for each penalty type. The table provides an overview of the frequency of each sanction associated with the corresponding ethical article. Each article includes a series of subsections, which were grouped together to facilitate interpretation and visualization of the data.

**Figure 2:**
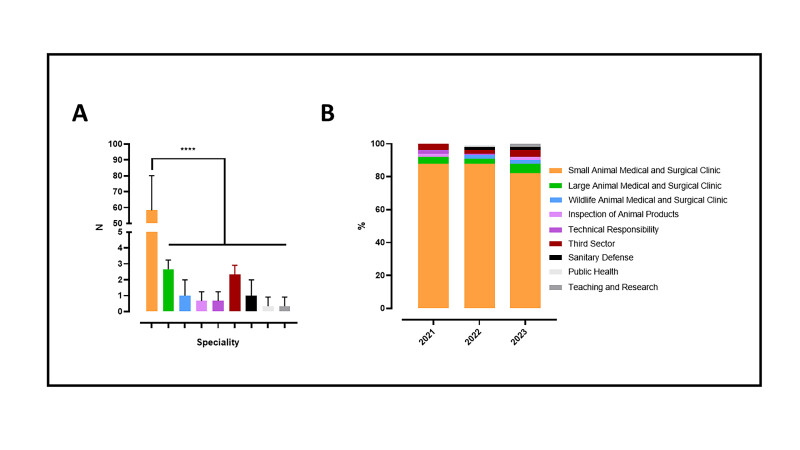
Analysis of the specialties involved in the processes.

Note: A- Total number of complaints by specialty (n=208). B- Annual distribution of complaints by specialty.

Figure 2A depicts the opened proceedings related to small animal clinics and surgery care, followed by large animal clinics and surgery, and then wild or exotic animal clinics and surgery.

This distribution may reflect the predominance of certain fields of veterinary medicine in daily clinical practice. Although no major changes were observed during the evaluated period, there was a noticeable trend toward an increase in complaints related to large animal clinics and surgery by 2023 (Figure 2B). Other areas such as inspection of animal products, technical responsibility, third sector, sanitary defense, public health, and lastly teaching and research were also represented throughout that period. These latter categories, although proportionally less frequent in the dataset, often involve different types of complainants — such as from veterinarians themselves or public agencies — rather than animal owners, which may influence reporting patterns and evaluations in ethical proceedings.

**Figure 3:**
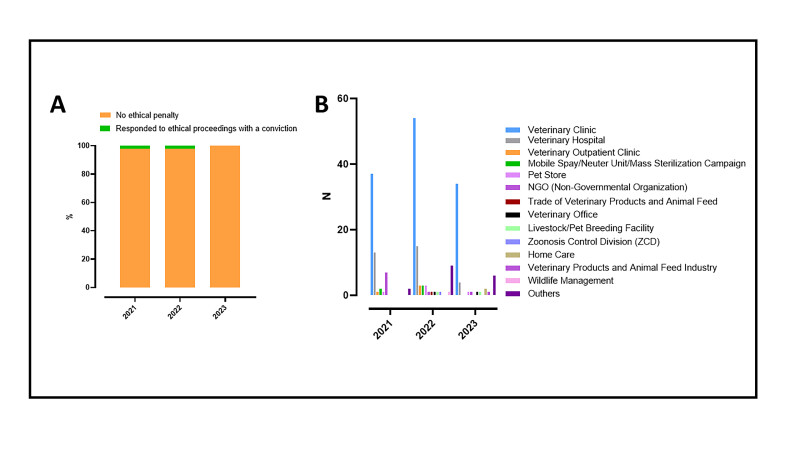
Presentation of whistleblowers and types of complaint.

Note: A- Distribution of whistleblowers by year. B- Types of infractions reported each year.

Figure 3A presents a stacked bar chart showing the percentage distribution of complaint or notification sources from 2021 to 2023. The categories include: CRMV-SP ex officio, veterinarians, the Ministry of Agriculture and Livestock, the police, animal guardians or owners, third sector entities, and others. Across all years, most notifications originated from animal guardians, reflecting the central role of client-veterinarian interactions in the initiation of proceedings. Additionally, there is a noticeable trend toward an increase in complaints filed by public institutions such as the police and the Ministry of Agriculture, as well as by veterinarians themselves, suggesting a growing awareness and engagement of professionals and regulatory bodies in upholding ethical standards. There was little participation from other categories, such as the police and the Ministry of Agriculture and Livestock, however, it varied from year to year. CRMV-SP Ex-Officio played a more relevant role in 2023 than in previous years. Figure 3B depicts the number of cases related to the different types of infractions or problems identified over the years. The main reason for this occurrence is associated with negligence, recklessness, or malpractice in the services provided, which remains the category with the highest number of records in all years analyzed.

The second most frequent infraction involves the failure to complete, improper completion, or missing documentation, which represents one of the main causes of ethical violations. This other category brings together a more diverse set of reasons, but with a lower incidence, even as it was present in all three years. In addition, failure to clarify the risks, limits, and consequences of procedures had significant records, especially in 2021. In general, there was a reduction in the total number of infractions from 2021 to 2023, especially in the categories of negligence, recklessness, or malpractice.

**Figure 4:**
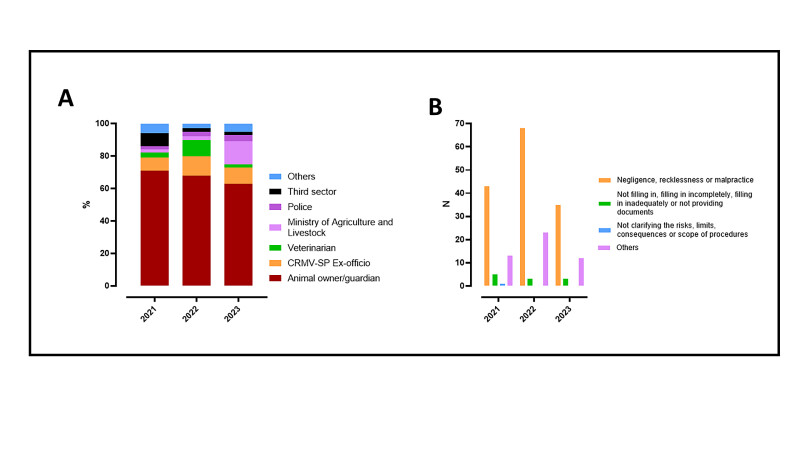
Presentation of repeat offenders and reported locations.

Note: A- Distribution of repeat offenders by year. B- Places where the incident that led to the complaint took place each year.

Regarding the existence of previous cases, the proportion of repeat offenders was low, accounting for 2% of the total in both 2021 and 2022, and 0% in 2023 (Figure 4A). When the activity of veterinarians (distributed by area) was evaluated, it was observed that in the majority of cases, the defendant worked in small animal veterinary clinics (Figure 4B).

**Figure 5:**
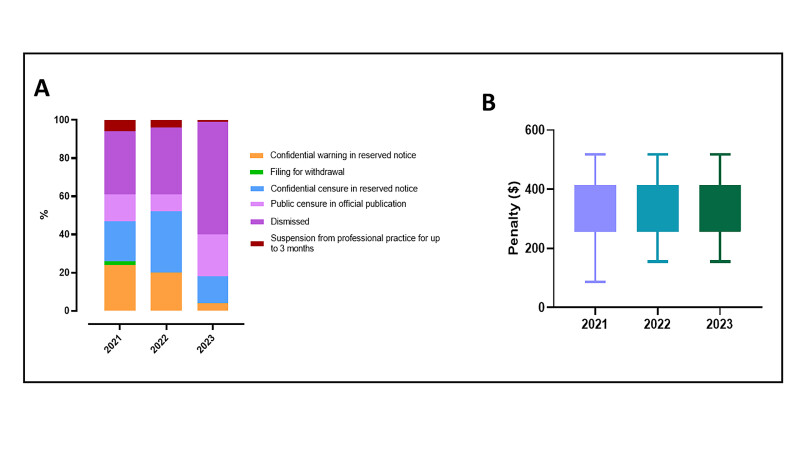
Determined penalties.

Note: A- Main types of penalties. B- Monetary amounts imposed on defendants. Details of the articles infringed compared with the penalties are shown in Table 1.

Figure 5 shows that 124 cases resulted in convictions (59.61% of the total), with confidential warnings being the most frequently applied penalty, as shown in Figure 5A. In 2023, there was an increase in the number of case dismissals and a decrease in the application of confidential warnings as penalties. As a result, censures and dismissals became the most prevalent outcomes.

The analysis of ethics-based cases against veterinarians revealed different types of penalties applied based on the articles that were infringed. A total of 208 cases were analyzed, including warnings, reprimands, suspensions, and dismissals. One denied case has been removed from this statement.

Among the 208 cases analyzed (excluding the denied case), the most frequently applied penalty was confidential censure in a reserved notice, accounting for 50 cases (24%). This penalty was most often associated with Article 8, particularly with its subsections I-b (12 cases) and I-c (9 cases). The second most common penalty was confidential warning in a reserved notice, applied in 37 cases (18%), mainly linked to Article 9, with a predominance of item I (16 cases), followed by item II (12 cases) and item III (5 cases). Notably, 7 professionals (3.4%) received penalties referencing all three items of Article 9 simultaneously (I + II + III), indicating more complex or recurrent infractions. Official publication of public censure was applied in 28 cases (13%), also with a strong association with Article 8, especially subsection I-a (15 cases) and I-b (5 cases). Suspension from professional practice for up to three months occurred in 8 cases (4%), predominantly due to infractions of Article 8, notably subsections I-a and I-b, which together accounted for 6 out of the 8 suspensions. Finally, 84 cases (40%) were judged unfounded, resulting in no penalties.

## Discussion

Statistical surveys of proceedings before professional councils are common in the field of human medicine (Harbitz et al., 2021). This paper presents a survey of veterinary medicine and analyzes 208 ethical cases before the São Paulo State Veterinary Medicine Council between 2021 and 2023. The cases that resulted in convictions were 124 (59.33% of the total), identifying the profile of the professionals denounced, the main reasons for the complaints, and characterizing the case law in the form of established penalties.

The difference between the number of complaints between 2021 and 2023 shows that there is indeed a trend and an increase in the number of ethics-based complaints in the São Paulo regional council, which was reported that in 2021 there were 84 complaints against veterinarians, following with 119 complaints in 2022, and 119 complaints in 2023. This can be explained by the fact that there has been a clear abrupt change in recent years, which is represented by the knowledge of the rights of those responsible for animal rights, reflecting the importance of animals in homes (Pugliese et al., 2019). The strengthening of the bond between humans and animals has significantly transformed the veterinary profession.

Today, pets play a central role in many people’s lives, serving as companions, sources of emotional support and, in some cases, replacing the presence of children in families. This paradigm shift has intensified guardians’ expectations in relation to veterinary care, demanding a high standard of care and professionalism, prompting people to be more active in seeking the judiciary and professional councils for damages caused by veterinarians through service failures (Pazó & Heancio, 2014; Silva, 2019).

The study of ethics is important from the functional aspect of society. In addition to establishing rules for peaceful coexistence between people, it guides professionals in respecting the interests of individuals (Monte, 2002). This work serves as a reference to highlight the scarcity of this type of study, with the purpose to contribute to the administrative area. Knowledge is fundamental in order to reduce the number of complaints and infractions regarding veterinary practice with state councils across the country.

The data reveals that the profiles of professionals who are denounced corresponds to that of veterinarians who have been trained for less time. This finding corroborates what was described by Tostes in 2024, in his chapter on deontology in the book Tratado de Medicina Veterinária Legal. He also emphasizes that in this context, not only is the lack of refined technique an influence on this result, but also the lack of knowledge by some veterinarians of the Code of Ethics that governs their profession. The lack of knowledge on these points can be attributed, in part, to shortcomings in educational institutions, which have historically inadequately emphasized the rights and duties of professionals during academic training. Although subjects such as Deontology and Ethics are present in some curricula, their approach is not always in-depth or practical enough to prepare future professionals for the ethical challenges of everyday life.

According to data from the Federal Council of Veterinary Medicine (CFMV), as of the time of this article's publication, Brazil has approximately 536 authorized undergraduate veterinary medicine programs, of which around 476 are actively operating. Brazil is one of the countries with the highest number of veterinary schools worldwide (Wouk et al., 2022). In addition, the reduction in compulsory workload and clinical practice during undergraduate courses compromises the development of technical skills and professional safety of recent graduates, leaving them more susceptible to mistakes that can culminate in ethical and civil complaints.

Modern veterinary medicine requires not only clinical competence but also interpersonal skills, such as assertive communication, crisis management, and emotional control, especially in the face of increasingly enlightened and demanding guardians. Many young veterinarians enter the market without adequate training in soft skills, which makes them vulnerable to conflicts with clients, poor handling of critical cases, and difficulties in making decisions (Fogle et al., 2021; Moraes et al., 2014).

According to the figures in this study, the majority of complaints registered with the Regional Council of Veterinary Medicine of São Paulo (CRMV-SP) came from animal owners who were dissatisfied with the care they received. Most of these complaints involve allegations of negligence, recklessness, or malpractice on the part of professionals, followed by failure to provide information. It is important to emphasize that the perception of the error and the issue to which it is related is poorly understood by veterinarians (Oxtoby et al., 2015).

The creation of the Admissibility Commission in 2020 by the CRMV-SP, with the purpose of analyzing preliminary complaints against veterinarians, represented a significant advancement in the management of ethical-professional proceedings within the state. This measure, implemented in accordance with CFMV Resolution No. 1330/2020 (Brasil, 2020), established a consultative technical chamber responsible for assessing whether there are sufficient indications of ethical infractions to justify the opening of a formal process. It is important to note that the establishment of such a commission is not mandatory for all Regional Councils and that its recommendations are not binding. The final decision on whether to initiate or archive a case remains under the exclusive authority of the Council’s president. The data analyzed shows that, since the implementation of this commission, there has been a significant reduction in the number of ethical cases brought, as well as a drop in cases considered unfounded, compared to data from 2018 published by CRMVSP. Our study showed that there is a current conviction rate of 40% for unfounded cases, and before the commission, it was approximately 60% (CRMVSP). This impact suggests that careful screening of complaints directly contributes to greater efficiency in professional inspection, preventing unfounded accusations from overloading the disciplinary system, optimizing resources, and playing a crucial role in protecting veterinarians from the emotional and financial strain of responding to unsubstantiated cases.

The initiation of an ethical process, even when unfounded, imposes legal costs on the professional, as well as significant psychological impacts, which can damage their reputation and professional stability. Thus, adopting this preventive mechanism not only improves procedural justice within veterinary medicine, but also strengthens the credibility of the inspection system, ensuring that only legitimate and duly substantiated complaints are taken forward for analysis by disciplinary bodies.

Finally, it should be noted that the data obtained has been statistically analyzed, allowing not only a survey of the main reasons for complaints but also an assessment of the uniformity of the penalties applied. This identification of patterns will allow for a better understanding of the challenges faced not only by professionals, in order to help prevent new occurrences and bring clarity and objectivity to the attitude that veterinarians should take when faced with an ethical complaint, but also to provide an opportunity for the council to take a more critical look at the processes and everything that surrounds them, including the sometimes-arduous mission of judging.

Veterinary clinics are the establishments most frequently reported to the Regional Council of Veterinary Medicine, a fact that can be analyzed from several perspectives. First, it is essential to consider the significant number of clinics operating in Brazilian states, which significantly increases the incidence of complaints directed at these establishments. In addition, clinics deal with a wide range of cases, from routine consultations to more complex procedures, which can increase the likelihood of disagreement between animal guardians and professionals. The lack of standardization in clinical protocols; failures in communication with owners; and difficulties in clinical, technical, and document management can contribute significantly to the increase in complaints, often related to professional conduct, quality of care, and contractual issues between veterinarians and clients.

Veterinary hospitals, on the other hand, rank second in complaints, which may be related to the nature of the care provided in these establishments. Unlike clinics, hospitals deal with more complex cases, receive referrals, and offer a wider range of services such as laboratory tests, diagnostic imaging, and hospitalization. This variety of procedures increases the hospital’s exposure to questions from guardians, especially when there are disagreements about prognosis, clinical evolution, or adopted therapeutic approaches. In addition, the hospital structure often involves multiple professionals caring for the same patient, which can lead to complications in communication and, consequently, dissatisfaction of animal guardians who are looking for more direct, clarifying, and individualized answers.

The data not only highlights the failure to provide veterinary medical services but also infractions related to documentation, such as inadequate completion or failure to provide mandatory documents such as medical records and reports. This problem reflects the importance of knowledge regarding regulations and the need for greater rigor in document management within veterinary clinics and hospitals. Failure to clarify the risks and limits of procedures through medical records and informed consent forms also appears frequently, which may indicate failures in the duty to inform and in communication between professionals and guardians, resulting in insecurity and subsequent questions about clinical conduct and the duty to inform.

Another concern is the occurrence of disparaging comments among veterinary colleagues, demonstrating a major ethical challenge within the professional class itself. The Code of Ethics for Veterinary Medicine reinforces the need for mutual respect, and does not publicly expose the conduct of other professionals without technical grounds. However, this type of infraction persisted. The increase in complaints between colleagues shown in this survey may be linked to an environment of fierce competition, or even a lack of professional maturity in dealing with technical differences in an ethical manner.

Regarding infractions, the most frequently violated articles included Article 9, which addresses failures in the provision of professional services. It is important to note that this and the other articles encompass multiple sections and subsections; however, for the purpose of clarity and data presentation, the results were grouped and reported at the article level. Article 10 - Also with significant records, possibly related to inadequate compliance with technical and regulatory standards. Articles 1, 2, and 3 present some penalties related to fundamental principles of the profession. This shows that diligent technique is a prerequisite for the prevention of ethical proceedings, as failure to provide services through malpractice, negligence, and recklessness proved not only to be the main motivation for complaints, but most convictions were in line with these motivations resulting in the penalizing of veterinarians, as well as in the majority of infringements of Article 9.

There is also a clear need to discuss this issue so that not only do veterinarians know how to protect themselves through this knowledge, but they also know what to expect and how to act in the event of a complaint.

The figures also show that many veterinarians are convicted of not properly filling in documents, such as medical records, or not handing them in. This situation is recurrent in routine forensic and technical assistance, where medical records are often absent for forensic analysis or incomplete records.

Among the cases in which punishment was handed down, confidential warnings (37), reprimands (50), and fines stood out, suggesting that most of the infractions were considered mild or moderate. Only a small number of cases resulted in the suspension of professional practice (8), indicating that serious infractions and recidivism are less frequent. No robust study has been conducted on these details in veterinary medicine. This data is consistent with studies that analyze ethical-professional processes in other areas of health, such as human medicine, where the majority of decisions also result in mild-to-moderate penalties, with confidential warnings and censures being the most prevalent (Coltri, 2024). The data shows that, as in human medicine, there has been a trend towards stricter penalties in recent years.

Finally, the convictions clearly reflect a failure in the duty to inform, which stands out as one of the main reasons for conflict. This failure is also evident in the most recurrent articles infringed as the basis for the penalties imposed.

## Conclusion

There is a lack of studies and literature on this subject, and this work is essential to contribute to the scarce scientific research and discussion on this subject in veterinary medicine. Although knowledge of the Code of Veterinary Medical Ethics is a professional obligation, as established in Article 17, some of the proceedings suggest that this requirement is not always met in practice. This highlights the need for continuous professional education, especially regarding the main types of infractions, the identity of whistleblowers, and the motivations behind complaints submitted to the professional council. Penalties should also be applied in a consistent manner, in accordance with the nature of the infractions.The transparency of this information is essential for the veterinary profession to take adequate precautions and know what to expect from this process, promoting closer, more transparent, ethical, and safer professional practice.
